# Atlantic cod (*Gadus morhua*) larvae are attracted by low-frequency noise simulating that of operating offshore wind farms

**DOI:** 10.1038/s42003-023-04728-y

**Published:** 2023-04-12

**Authors:** Alessandro Cresci, Guosong Zhang, Caroline M. F. Durif, Torkel Larsen, Steven Shema, Anne Berit Skiftesvik, Howard I. Browman

**Affiliations:** 1 Institute of Marine Research, Ecosystem Acoustics Group, Austevoll Research Station, Sauganeset 16, N-5392 Storebø, Norway; 2 Institute of Marine Research, Ecosystem Acoustics Group, Nordnesgaten 50, 5005 Bergen, Norway

**Keywords:** Animal migration, Behavioural ecology

## Abstract

The number and size of offshore wind (OW) turbines is increasing rapidly. OW turbines produce continuous, low-frequency noise that could impact marine fish dispersing/migrating through the facilities. Any such impact would be relevant for larval stages, which have limited possibility to swim away from OW facilities. If directional movement of fish larvae at sea is impacted by low-frequency continuous sound is unknown. We observe the behavior of Atlantic cod larvae (*N* = 89) in response to low-frequency sound while they are drifting in a Norwegian fjord inside transparent drifting chambers. We transmit 100 Hz continuous sound in the fjord, in the intensity range of OW turbines’ operational noise, and measure the sound pressure and 3-D particle motion. Half of the larvae (*N* = 45) are exposed to low-frequency (100 Hz) continuous sound, while the other half (*N* = 44) are observed under the same conditions but without the sound. Exposure does not affect the routine and maximum swimming speeds or the turning behavior of the larvae. Control larvae orient to the northwest. In contrast, exposed larvae orient towards the source of low-frequency sound and particle motion. This provides a basis to assess how OW might impact dispersal in this species.

## Abstract (web summary)

An open water experiment in the North Sea, using drifting behavioral chambers, demonstrates that cod larvae orient to the source of low-frequency underwater noise at the level generated in the proximity of an offshore wind turbine.

## Introduction

The number and size of offshore wind facilities is increasing rapidly to meet the demand for renewable energy^1,2^. In Norwegian waters, more than 1500 offshore wind turbines covering an area of >12,000 Km^2^ will be installed by 2040. The objective is to produce at least 30,000 MW (https://www.nrk.no/norge/vil-produsera-nesten-like-mykje-havvindkraft-som-dagens-vasskraft-1.15962706). Analogous expansions in offshore wind facilities are planned in other countries, particularly in USA, UK, and China. This rapid expansion of the footprint of offshore wind facilities raises questions about their potential impacts on marine animals and ecosystems.

When wind turbines operate, they produce underwater noise that can reach distances of kilometers from the sound source^3^. The intensity of the noise increases with wind speed and size of the turbines^4,5^. There are major gaps in our knowledge of the responses of marine animals to the sound and vibration caused by operating turbines^6^.

When operational noise propagates in the marine environment, it becomes part of the soundscape: a collection of biological, geophysical, and anthropogenic sounds that vary over space and time^3,7,8^. The continuous sound produced by wind turbines can modify the soundscape at distances of kilometers and over long periods of time (decades)^8,9^. Thus, there is concern that the introduction of continuous noise from large-scale offshore wind facilities will affect the behavior of fish, invertebrates, and marine mammals that use sound for mating, foraging, and movement-orientation^8,10^. The effects of exposure to the sound produced by offshore wind turbines may be particularly relevant during the dispersing early life stages, as these contribute to determining recruitment success, population size, and distribution of the adults. Any influence that operational noise from offshore wind turbines might have on larval dispersal ecology^11–13^ could have possible downstream population-level effects.

During the dispersal phase of their life history, some fish larvae are capable of using sound in the low-frequency domain for orientation towards suitable habitat and settlement areas^14,15^. This type of directional orientation response is guided by sensing both the sound pressure and particle motion associated with the sound, which provides directional information relative to the source^16^. Although considering particle motion in studies on fish response to sound is essential, it remains challenging to measure in situ and, therefore, has rarely been measured, and almost never in coordination with behavioral observations^16^. Describing how fish (larvae, juveniles, and adults) respond to the particle motion produced by operating wind turbines is one of the main research priorities in the context of understanding the potential impacts of offshore wind facilities on wild fish populations^6^.

Operational noise produced by offshore wind turbines (OWTs) is in the low-frequency range (<700 Hz), with most of the energy concentrated between 2 and 200 Hz^4,17^. This frequency range overlaps with that used by fish for communication, mating, spawning, and spatial movement^5,8,17–19^, and by fish larvae for movement and orientation during dispersal^14,20–22^. However, there is no information on the impacts of low-frequency continuous sound in the frequency and intensity domain of that produced by OWTs on the behavior of marine fish larvae that reside in, or disperse through, areas where offshore wind farms operate.

Atlantic cod is an ecologically and commercially important species in the North Sea and the Norwegian Sea^23,24^. Sound plays an important role in the ecology of cod. They produce sounds during mating and spawning, and use sound for spatial orientation to aggregate during spawning events^18,25^. This species can perceive low-frequency sound (30–500 Hz), and their larvae drift in areas of the North Sea where offshore wind facilities will be placed (https://www.equinor.com/no/what-we-do/floating-wind.html). Thus, cod larvae that will drift in the proximity of, or through, large-scale offshore wind facilities will be exposed to the operational noise produced by OWTs. Whether cod larvae - or the larvae of any other species - will be impacted by that noise is unknown.

**Fig. 1:**
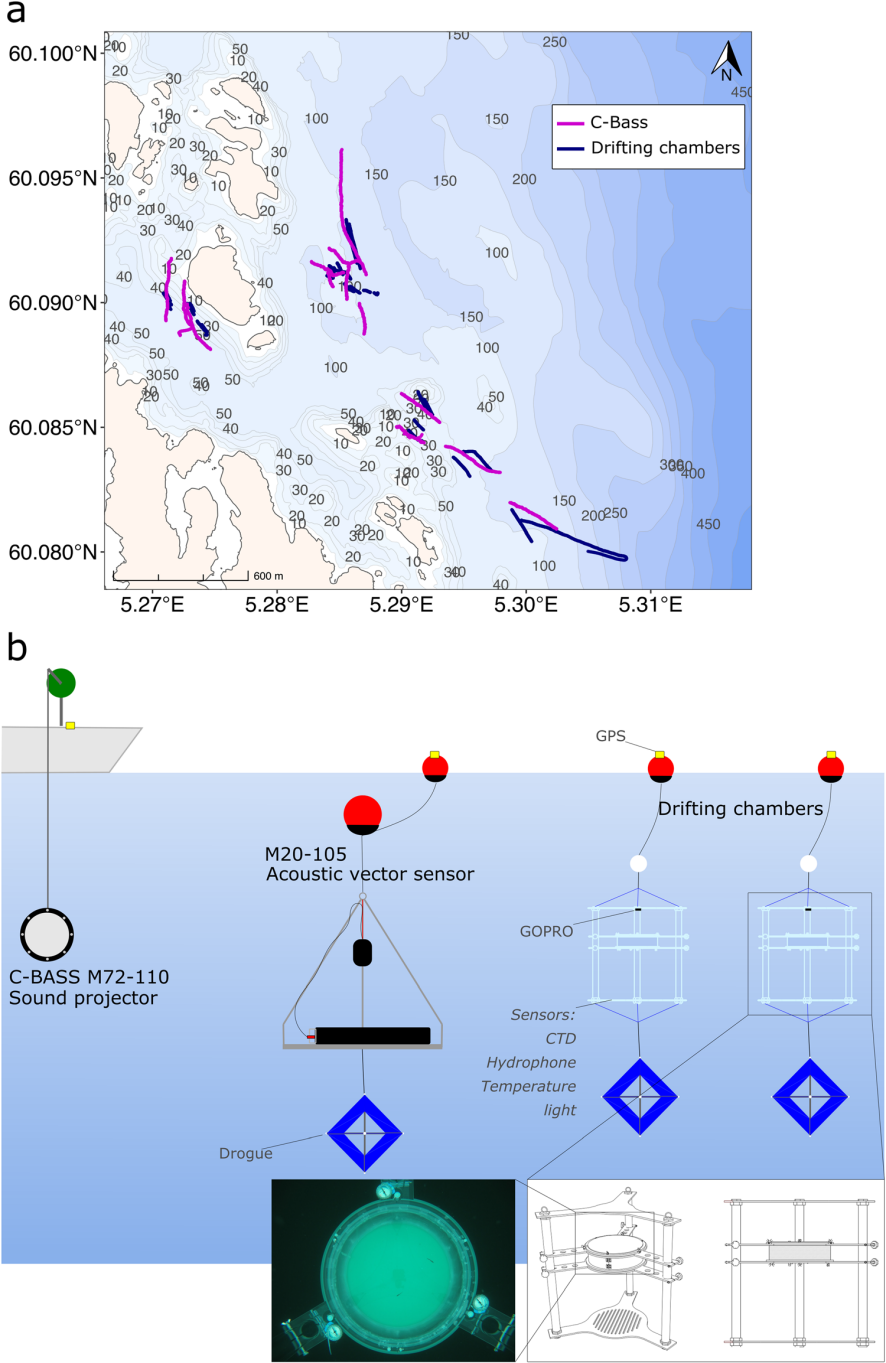
Area where the experiments on Atlantic cod (*Gadus morhua*) larvae were conducted and experimental setup. **a** Map with the location where experiments were performed. The map shows the coastline and bathymetry (coastline and bathymetry layers from the open-source repository www.kartverket.no). Magenta lines are the GPS tracks of the C-Bass sound projector and navy-blue lines are the GPS tracks of the drifting chambers containing fish larvae. All of the tests were performed at a distance of <200 m between the C-Bass and the chambers. During any given test, both chambers were deployed at the same time, each one containing two cod (*Gadus morhua*) larvae. In total, we video-recorded the behavior of 89 cod larvae. **b** Schematic diagram of the experimental design. The sound projector (C-Bass M72-110, GeoSpectrum Technology Inc. (GTI)), the acoustic vector sensor (M20-105, GTI), and the drifting chambers were all outfitted with drogues and drifted independently. The projector, the acoustic vector sensor, and the circular arena of the drifting chambers were all at 5 m depth. The chambers were equipped with an array of sensors (CTD, hydrophone, temperature, light) placed on the bottom grid of the acrylic frame. The bottom-right white box shows the technical drawings of the drifting chambers, with a picture showing the view from the GOPRO mounted on the frame that recorded the videos used for the tracking of the larvae, which are clearly visible.

In this study, we used a unique approach to assess the response of Atlantic cod larvae (*N* = 89) to continuous low-frequency sound in the frequency and intensity range produced by operating OWTs. Cod larvae were exposed to low-frequency sound while they were drifting in transparent behavioral chambers at sea (Fig. 1) and their behavior was video-recorded during 15 min trials. During these observations, continuous low-frequency sound was produced using a submersible high-output low-frequency sound projector. The particle motion associated with the low-frequency sound was sampled and characterized using a state-of-the-art 3D underwater acoustic vector sensor (AVS). The swimming, turning, and orientation behavior of the cod larvae was extracted from the videos and used to assess whether they were modified by the low-frequency sound.

**Fig. 2:**
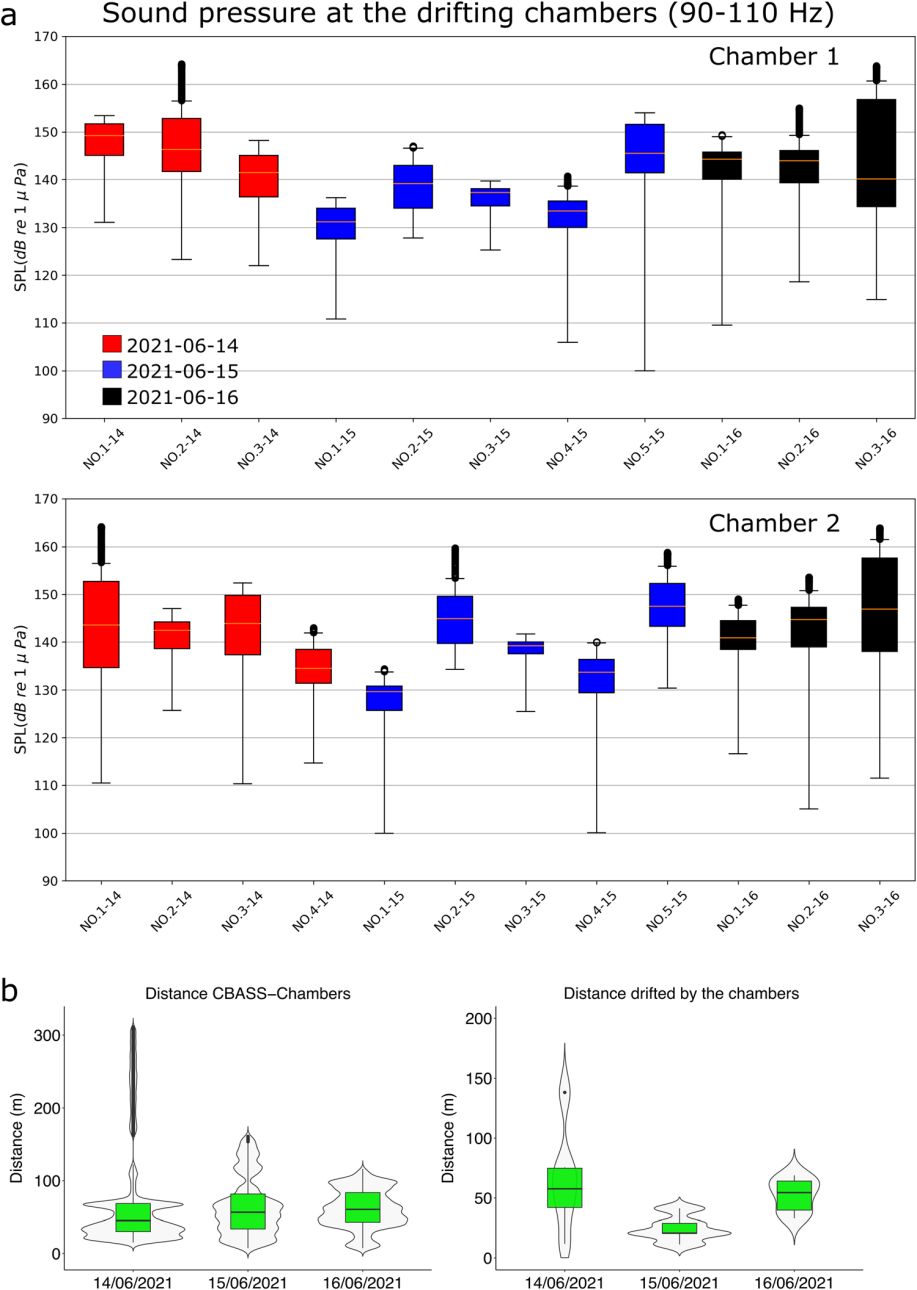
Sound pressure level (SPL) at 90–110 Hz received at the drifting chambers enclosing cod larvae (*Gadus morhua*), and data from GPS tags from the sound source and the drifting chambers. **a** Overview of the SPL (*dB re* 1 μPa) delivered at chamber 1 and chamber 2 during each of the 12 transmissions. There are 11 boxplots for chamber 1 and 12 for chamber 2 because one transmission (NO.4-14) was performed with only one chamber at sea (chamber 2) on 2021-06-14. The SPL boxplots are computed on a linear scale and visualized on a logarithmic scale. Boxplots show minimum, 25th percentile, median, 75th percentile, and maximum values. *N* = 700 for each boxplot (sampling frequency of 1 Hz). Boxplots are colored according to the date of the experiment. **b** Boxplot of the relative distance between the boat carrying the sound source (C-Bass) and the drifting chambers, and boxplot graph of the total distance that each chamber drifted, moved by the current, during each deployment. Boxplots are displayed for each day of deployments.

Half of the cod larvae (Exposed larvae; *N* = 45) were exposed to the low-frequency continuous sound while they were drifting at 5 m depth in a Norwegian fjord (Bjørnafjorden, Fig. 1a). The other half of the cod larvae (Control; *N* = 44) were tested under the same conditions, but in the absence of low-frequency continuous sound (the sound projector was switched off). The whole experiment was conducted in situ, with the low-frequency noise added to the natural soundscape in the fjord. Both the acoustic equipment (sound source, AVS, and hydrophones) and the two drifting behavioral chambers in which the cod larvae were observed (Fig. 2a) were deployed as independent, free-drifting units (Fig. 1b). The drifting equipment was fitted with a drogue to ensure that they would drift with the current in the fjord’s surface layer. All units (sound projector, AVS, and the two drifting chambers) were equipped with GPS loggers, so that their position and their relative distance were logged throughout the experiment (Fig. 1b).

Using this approach, we tested the null hypothesis that noise in the intensity and frequency range of the operational noise generated by OWTs had no effect on the swimming, turning, or orientation of Atlantic cod larvae.

## Results

### Sound exposure

There were 12 sound transmissions during the three days of experiments on cod larvae, during 14–16 June 2021 (Fig. 2a and Table S1). All of the transmissions were conducted at a distance of less than 320 m between the sound source and the drifting chambers (Fig. 2b). The two drifting chambers were always within 70 m of each other (Fig. 2b).

The median sound pressure level (SPL) at 100 Hz delivered at the drifting chambers (Fig. 2a) during the sound transmissions throughout the experiment was $139.5\,(11.8)\,{dB}\,{re}\,1\,\mu {Pa}$ (median (interquartile range—IQR)). The overall SPL delivered at chamber 1 was $139.3\,(11.9)\,{dB}\,{re}\,1\,\mu {Pa}$ and it was not significantly different (*W* = 81, *p* = 0.64) compared to the median SPL of $139.6\,(11.6)\,{dB}\,{re}\,1\,\mu {Pa}$ delivered at chamber 2 (Fig. 2a). The SPL varied between the transmissions as the experiment was conducted in situ. Thus, as expected, the sound propagation conditions varied with time due to the drifting of the sound source and receivers, the bathymetry, and weather conditions.

**Fig. 3:**
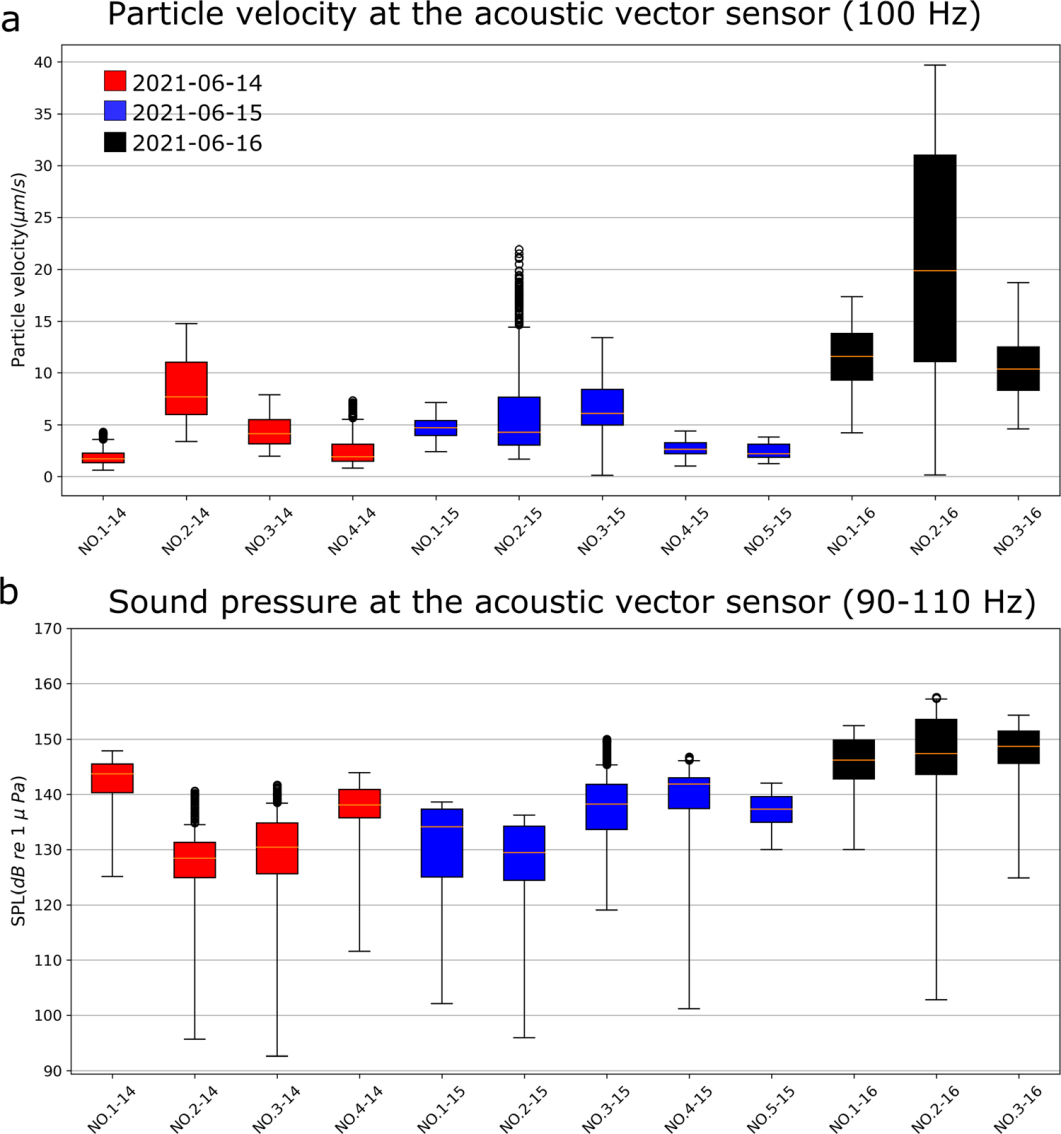
Particle velocity and sound pressure level (SPL) received by the acoustic vector sensor M20-110 that was deployed during the exposure of cod larvae (*Gadus morhua*) to a continuous 100 Hz sound. **a** Particle velocity *(*μm/s) at 100 Hz measured by the M20-110 vector sensor during each sound transmission. **b** SPL (dB re 1 μPa) at 90–110 Hz measured by the M20-110 omnidirectional channel during each transmission. *N* = 700 for each boxplot (sampling frequency of 1 Hz). Boxplots are colored according to the date of the transmissions.

The particle velocity and SPL at the acoustic vector sensor also varied within each transmission and between transmissions (Fig. 3a, b). For most transmissions, the particle velocity was $< 15\,(\mu m/s)$ (Fig. 3a). The seemingly anomalous increase in particle velocity during the transmission *N.03-16* (Fig. 3a) was caused by the AVS drifting close to the sound projector (<25 m). However, this does not reflect the delivered sound pressure at the drifting chambers during transmission *N.03-16* (Fig. 2a), as the chambers were drifting independently of the acoustic vector sensor, and they were further away from the sound source (Fig. 2a). The median SPL measured by the omnidirectional channel of the acoustic vector sensor was $137.9\,(12)\,({dB}\,{re}\,1\,\mu {Pa})$, which was similar to that measured by the hydrophones mounted on the drifting chambers (Figs. 2a,3b).

**Fig. 4:**
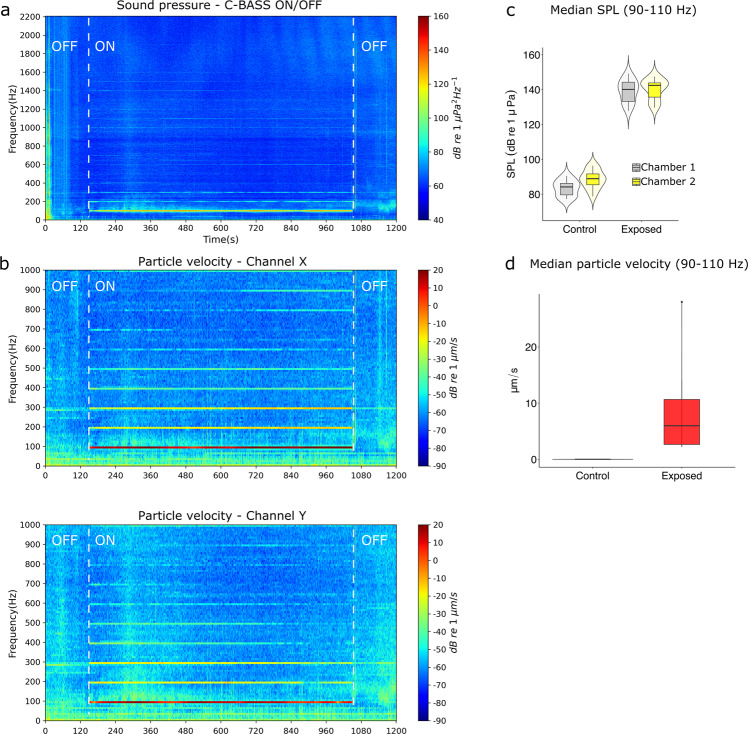
Spectrograms of the acoustic pressure (received at chamber 2) and particle velocity across frequencies during a 15 min-long sound transmission at 100 Hz (transmission NO.4-15). The particle velocity was measured by the M20-110 acoustic vector sensor and the acoustic pressure was measured by the AMX-Remora hydrophone mounted on the chamber. Both sensors were drifting at 5 m depth in the fjord during the experiment. **a** Spectrogram of power spectrum density during a time period with the sound projector switched OFF, then switched ON (dashed white line) and then OFF again (dashed white line). The processing parameters are segment time length of 10 s, 80% overlap, fast Fourier transform length of 4410, and Hann window. **b** Horizontal particle velocity (*dB re* 1 μm/s) during one of the tests, with the sound projector switched OFF and then switched ON. The particle velocity is displayed both from the X channel and the Y channel of the M20-110 sensor. The velocity is calculated every second with no overlap. **c** Median sound pressure level (SPL) at 100 Hz received by the chambers when the sound projector was switched OFF (Control) (*N* = 11 for chamber 1, *N* = 11 for chamber 2) and when the sound projector was switched ON (Exposed) (*N* = 11 for chamber 1, *N* = 12 for chamber 2). **d** Median particle velocity measured by the M20-110 when the sound projector was switched OFF (Control; *N* = 11) and when the sound projector was switched ON (Exposed; *N* = 12).

The main low-frequency signal present in the soundscape in the fjord during the experiments was the 100 Hz sound signal produced by the C-Bass (Fig. 4a, b). This was true both when considering SPL (Fig. 4a) and particle motion (Fig. 4b). The 100 Hz transmission also generated higher harmonics at 200, 300, 400 Hz, and so on (Fig. 4a, b). The continuous 100 Hz sound emitted by the C-Bass was stable throughout the 15 min-long transmissions during each test (Fig. 4a, b).

The SPL intensity delivered at the drifting chambers constituted an increase of 53.2 dB *re* 1 μPa at 100 Hz compared to the median 86.3 (7.0) dB re 1 μPa SPL at 100 Hz present in the background soundscape when the sound projector was not activated (Fig. 4c). The increase in SPL intensity at 100 Hz during the sound transmissions was significant (Wilcoxon test; *W* = 0, *p* « 0.05). The overall median background particle velocity at 100 Hz recorded by the M20-110 sensor was 0.03 (0.008) $\mu m/s$ (sound projector switched OFF), but it increased to $6.0\,(8.1)\,\mu m/s$ during sound transmission (Fig. 4d).

**Fig. 5:**
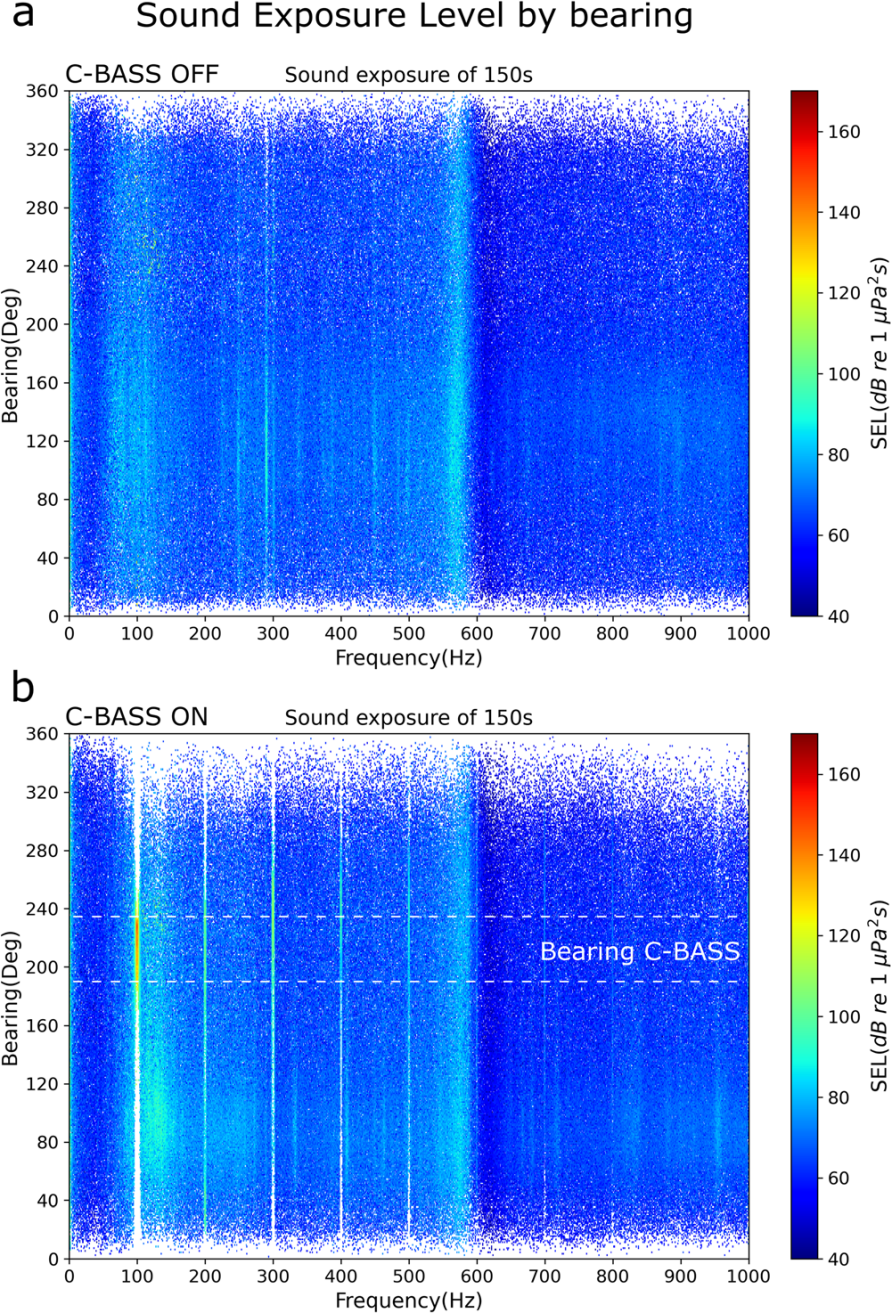
Sound exposure level (SEL) across frequencies and bearing measured by the M20-110 acoustic vector and sound pressure sensor. The bearing is of 1-degree resolution and the frequency is of 1 Hz resolution. The processing parameters are: segment time length of 1 s, 80% overlap, fast Fourier length of 20,000, and Hann window. **a** Ambient SEL (dB re 1 μPa^2^ S) from the acoustic pressure channel of the M20-110 displayed by bearing and frequency when the C-Bass speaker was turned off. **b** SEL from the acoustic pressure channel of the M20-110 displayed by bearing and frequency when the C-Bass projector was turned on. The horizontal dashed lines highlight the approximate bearing of the sound source (C-Bass) relative to the acoustic vector sensor.

The signal was highly directional (Fig. 5a, b). When the C-Bass was switched on, the most intense sound signal in the fjord was at 100 Hz, and its energy was concentrated in a narrow bearing in the direction of the sound source (Fig. 5b). Hereafter, when referring to particle velocity and sound direction, the bearing is defined as the angle of the incoming acoustic wave with the $x$-channel of the AVS pointing to the magnetic north. The angle is measured in degrees and calculated clockwise from the magnetic north.

### Swimming and orientation behavior of Atlantic cod larvae

The ambient temperature in the fjord at 5 m depth was 13.9 (0.13) °C and the salinity was 29.8 PSU. The total length of the cod larvae used in the experiments was 2.02 (0.28) cm (median (IQR)). Larvae from the Control group were not significantly different in size from those in the Exposed group (*W* = 1146, *p* = 0.20). Developmentally, larvae were at the postflexion stage^26^.

**Fig. 6:**
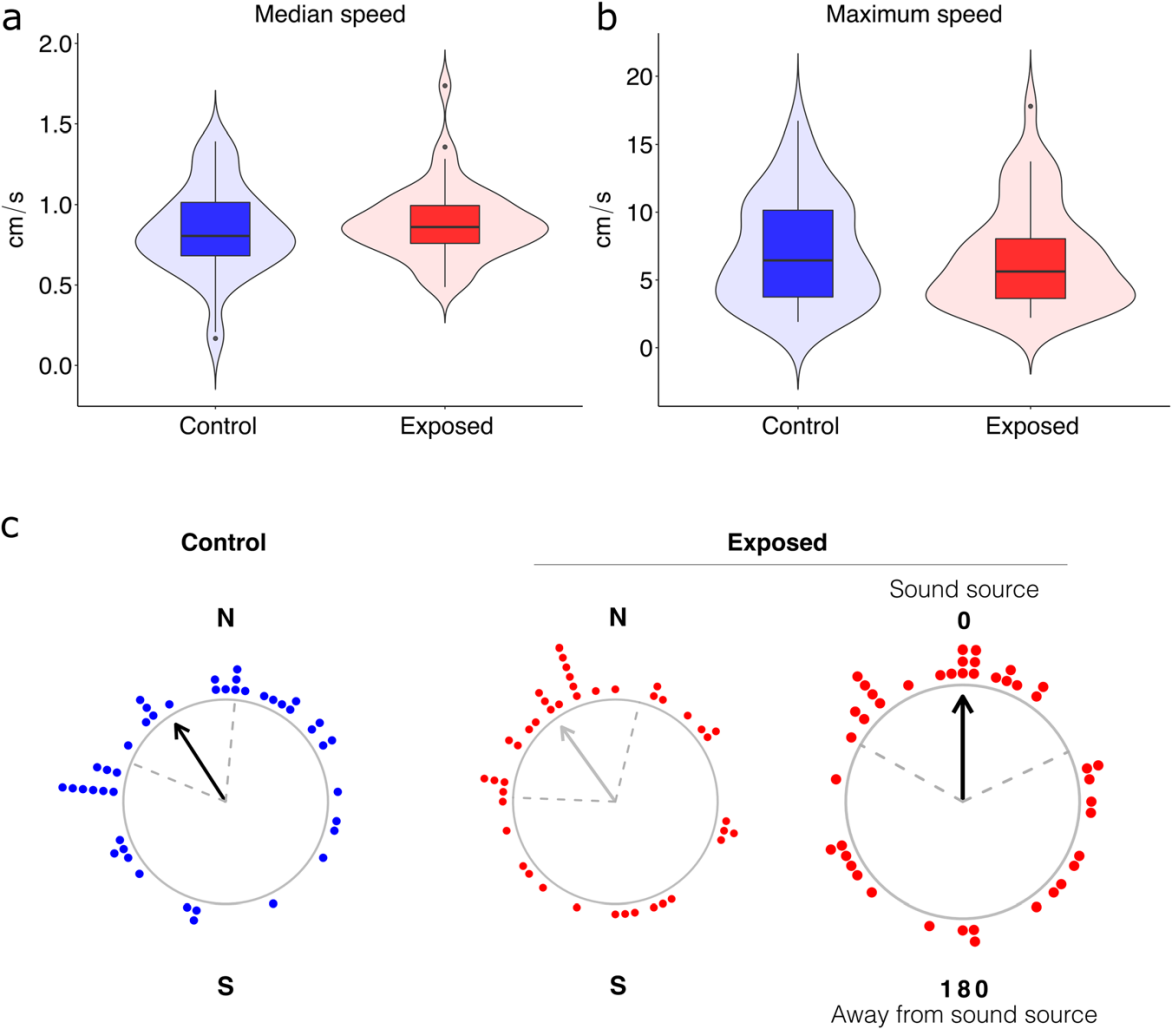
Swimming speed and orientation behavior of Atlantic cod (*Gadus morhua*) larvae in the drifting chambers. Boxplots show minimum, 25th percentile, median, 75th percentile, and maximum values, in the Control and Exposed groups. **a** Median routine swimming speed (Control: *N* = 44; Exposed: *N* = 45). **b** Maximum swimming speed (Control: *N* = 44; Exposed: *N* = 45). **c** Orientation of the significantly orienting larvae (larvae that showed an individual preferred orientation; *N* = 87) from the Control group (blue points; *N* = 44) and Exposed group (red points; *N* = 43) with respect to the magnetic north (N) and south (S), and orientation of the Exposed larvae corrected to the direction of the 100 Hz continuous sound source (C-Bass M72-110) (sound source). Correction to the direction of the sound source was performed on the tracks of each individual larva, every second, by using the relative direction sound source-chamber from the GPS data. Each point corresponds to the mean bearing of one cod larva in situ (averaged over 600 data points from the video tracks, Fig. S1). The arrow points toward the mean angle of all the individual bearings. The arrow is black when the orientation is significant (*p* < 0.05), as in the larvae from the Control group (Rayleigh’s test of uniformity, mean bearing in situ = 327°, *r* = 0.33, *p* = 0.006). The black arrow points towards the defined mean direction of 0° (sound source) in the plot showing the orientation of the Exposed larvae with respect to the sound source (Rayleigh test with the specified mean direction of 0°, *N* = 43, *r* = 0.23, *p* = 0.015). The arrow is gray in the plot of the orientation of exposed larvae with respect to the magnetic north as the orientation direction is close to significant (*N* = 43, mean direction = 324°, *r* = 0.26, *p* = 0.051). Dashed gray lines are the 95% confidence intervals around the mean.

The median routine swimming speed of cod larvae in the drifting chambers was 0.85 (0.30) cm/s. The routine speed did not differ between the Control and Exposed groups (*W* = 879, *p* = 0.37) (Fig. 6a). The larvae displayed a maximum speed of 5.98 (6.20) cm/s, which did not differ between groups (*W* = 1073, *p* = 0.50) (Fig. 6b). Control and Exposed larvae displayed the same turning behavior; the frequency distribution of the average turning angles (angle of change in swimming direction along a contiguous path) did not differ between Control and Exposed group (*D* = 0.086, *p* = 0.98).

In the Control group, all 44 cod larvae oriented (100% of the larvae had a preferred orientation angle inside the drifting chamber; Rayleigh test of uniformity applied to the track of each fish; *p* < 0.05; details on the analysis of orientation data are in section 4.4 and Fig. S1). In the Exposed group, 43 out of 45 larvae were oriented. The larvae from the Control group displayed an average orientation direction towards the northwest (*N* = 44, mean direction = 327°, *r* = 0.33, *p* = 0.006; Fig. 6c and Table S1). Exposed larvae displayed a preferred orientation direction toward the sound projector (Rayleigh test with the specified mean direction of 0°, *N* = 43, *r* = 0.23, *p* = 0.015; Fig. 6c and Table S2). Exposed larvae did not orient away from the sound source (Rayleigh test with the specified mean direction of 180°, *N* = 43, *p* = 0.90). The northwesterly orientation direction exhibited by Control larvae was still identifiable in the larvae from the Exposed group, but it was weaker and less accurate (*N* = 43, mean direction = 324°, *r* = 0.26, *p* = 0.051; Fig. 6c and Table S1).

## Discussion

Exposure to low-frequency (100 Hz) continuous sound affected the behavior of Atlantic cod larvae (*G. morhua*) swimming at sea in a wide fjord. The intensity of the 100 Hz sound signal to which cod larvae were exposed ($139.5\,(11.8)\,{dB}\,{re}\,1\,\mu {Pa}$) is in the intensity range of the continuous low-frequency noise measured in the proximity of operating OWTs. Wind turbines with nominal power of up to 6 MW produce broadband root mean square SPLs ranging from 129 to 166 dB re μPa 1 m (at 1 m from the turbine)^5^. The next generation of OWTs will have considerably higher nominal power (>10 MW) and are expected to produce continuous broadband sound pressure levels (SPL) of >170 dB re 1 μPa m (at close range)^5^. The 100 Hz tone used in this study was in the frequency range at which the operational noise from OWTs has the most energy (between 2–200 Hz)^4,17^. Therefore, the behavioral response of cod larvae to the sound signal used in this study is relevant in the context of assessing the possible behavioral impacts of operational noise from OWTs on cod larvae.

In the absence of continuous low-frequency sound, cod larvae oriented towards the northwest (Fig. 6c). Interestingly, this orientation direction is the same as that displayed in situ by the larvae of the closely-related gadoid Atlantic haddock (*Melanogrammus aeglefinus*)^27^. Among all larval behaviors, orientation direction and accuracy have the greatest impact on the drifting trajectory and fate of fish larvae at sea^13^. Thus, the northwest orientation direction displayed by cod and haddock larvae could have an important role in their dispersal and retention in the North Sea. The Norwegian Coastal Current (NCC) flows along the west coast of Norway in the form of a fast, narrow jet coming from the Baltic Sea^28^. This current follows the Norwegian coast from the Baltic all the way north to the Barents Sea. Larvae that adopt a northwest-directed orientation would be more likely to exit the western boundary of the NCC, avoiding being carried north towards the arctic. However, the adaptive significance of the larval orientation towards the northwest for this species is unknown. Further investigation using biophysical-coupled models is needed to understand the role and eco-evolutionary benefit of this behavior during dispersal.

Exposure to low-frequency continuous sound did not affect the swimming performance of cod larvae. However, exposure to low-frequency continuous sound affected their orientation. Exposed larvae swam towards (not away from) the 100 Hz sound. Although the northwest orientation displayed by Control larvae was still present, it was much weaker and less accurate, and was overridden by an orientation towards the source of the sound. Thus, in this experiment, exposure to continuous low-frequency sound such as that produced by OWTs overrode the direction towards which cod larvae naturally swim.

During the experiments, the low-frequency particle motion generated by the sound projector was the main directional sound signal present in the soundscape, concentrated along a narrow bearing towards the sound source (Fig. 5b). Cod are capable of resolving the direction of a sound source in both the horizontal and vertical planes^29^. They do that by integrating the detected plane of the particle motion with the gradient (from the source) in sound pressure^16^. This integration of acoustic signals could be achieved by using the otoliths and the swimbladder, both of which are developed in 2-cm long cod larvae such as those used in this study^26^. Thus, the orientation of cod larvae toward the sound source is likely a response to them detecting the low-frequency sound pressure and particle motion produced by the sound projector (Figs. 2–5). The results on cod larval behavior, and the concomitant measurements of particle motion reported in this study, highlight how research such as this is key to our understanding of the impacts of OWTs on the early life stages of fish^6^.

The attraction to low-frequency continuous sound reported in this study could be an evolutionarily advantageous behavior that evolved to increase the probability that late-stage larvae and juvenile cod settle in areas suitable for growth. At the transition between their pelagic and demersal life history periods, cod must find suitable settlement areas along the continental shelf and coast^30^, areas with complex substrates that are a source of low-frequency abiotic (from the action of waves and wind on the substrate) and biotic sounds^15,31,32^. In tropical areas, settlement-stage larvae are attracted in higher numbers, and with higher species diversity, towards the direction of reef sound^14,15^, especially under low-visibility conditions^20,33^. This attraction has also been reported in juvenile and adult stages of fish on reefs dominated by low frequencies (<1000 Hz)^33^. Cod could also be attracted by the low-frequency acoustic signature of settlement habitat, as this species is predominantly sensitive to low-frequency sound^18^, and lives in habitats where visibility can be very low.

The results reported in this study indicate that OWTs could have an attractive effect on drifting cod larvae. In the North Sea, swimming speeds of 3–6 cm/s can affect the dispersal of fish’s early life stages substantially, even in regions with strong currents^12^. Considering the size of future offshore wind facilities (thousands of square kilometers) and the effect that these will have on reducing wind forcing and changing ocean circulation, the swimming speed of cod larvae (0.8–6 cm/s), coupled with their orientation direction toward the sound source, could affect the dispersal trajectory of larvae that drift through or near a large-scale offshore wind facility. This attraction might act together with the artificial reef effect of the turbines and moorings in pelagic areas^34^ to modify the dispersal and spatial distribution of cod larvae at offshore wind farms. These possibilities must be assessed with further research to determine whether the larvae of other species respond as do cod larvae and to upscale these observations to population-level effects. The latter can be accomplished through scenario modeling using biophysical models of larval dispersal coupled with high-resolution oceanographic models and integrated with existing models of noise propagation from OWTs (e.g., ref. ^35^). Such an approach would allow the quantification of the role of orientation and swimming behavior on larval dispersal^12,13,36^ and the consequent impact of the disruption/modification of this behavior on larval drift following exposure to noise from OWTs.

## Methods

### Experimental animals

Atlantic cod broodstock were collected locally from the waters near Austevoll (60.085 N, 5.261 E), Norway, and used as the source of fertilized eggs. A mixture of fertilized eggs produced by a spontaneous spawning event from several males and females were placed into one 500 L tank at a density of 100 eggs/L. Water exchange was 4 L/min and tanks were on a 24 h light photoperiod under 2 × 25 w, 12 V halogen lamps. The larvae were reared in green water (Nannochloropsis, Reed Mariculture) at a temperature of 11–12 °C and a salinity of ca. 35 PSU. Larvae were fed on a diet of rotifers (*Brachionus sp*.) and natural plankton (mainly *Acartia* sp. nauplii) until 25 days post-hatch and then on *Artemia* and natural plankton (primarily *Acartia sp*.). Eggs hatched on April 6 and larvae started feeding on April 9, 2021. At the time of the experiment, larvae had a total length of 2.02 (±0.28) cm (median (IQR)) and were at the postflexion stage^26^. At least 1 h before being tested, the larvae were placed in a 5 L container filled with water from the rearing tank. The container was placed in a cooler box filled with seawater from the fjord surface layer. This allowed a slow transition from 12 °C (temperature of the rearing tank) to 13–14 °C (temperature of the fjord surface layer). A 3-cm-thick layer of neoprene surrounded the cooler box to provide shielding from ambient noise.

### Acoustic instrumentation

We deployed a low-frequency sound projector for acoustic transmission, and two hydrophones together with a 3D acoustic vector sensor (AVS) for field acquisition of acoustic data. The system used to transmit the sound signal consisted of a PC used as a signal generator, a low-frequency submersible acoustic projector (C-Bass M72-110, supplied by *GeoSpectrum Technology Inc*. (GTI), Canada), a power supply, and an M620 power amplifier supplied by GTI. The hydrophones used in this study were AMX-Remora hydrophones supplied by *Loggerhead*, USA (calibrated in February 2021). Hydrophones were mounted on the drifting behavioral chambers (Fig. 1), and used throughout the duration of the experiment to monitor the sound pressure that was received by the fish larvae swimming in the chambers.

Particle motion was measured and monitored throughout the experiment using the AVS M20-105 from GTI. The M20-105 was connected to an M209 data logger, a deployment frame, and a battery pack. The data logger acquired four channels of acoustic data simultaneously: the directional *x, y*, and *z* channels, and one omnidirectional acoustic pressure channel. The M20-105 sensor is also equipped with an internal magnetic compass and tilt sensors, which provide the heading and tilt of the acoustic vector sensor. Compass and tilt data are logged into the data logger in synchrony with acoustic data acquisition. The M20-105 was calibrated by GTI in Feb. 2021.

### Behavioral drifting chambers and tests on larval behavior in situ

The behavioral drifting chambers used in this study are a modification of the Drifting In Situ Chamber (DISC)^37,38^, which has been used in many studies on larval orientation and swimming behavior in situ^27,39–43^. The device consists of an acrylic structure, including a cylindrical chamber into which fish larvae are introduced and their behavior observed. The drifting chambers are designed so that the whole system drifts together with the current such that the larvae enclosed in the chambers drift with the current at the same speed. The bottom and top of the chamber are rigid and made of transparent acrylic, while the walls are made of mesh (500 μm mesh size). The mesh allows sound to pass through the chamber and for water and dissolved gas exchange, ensuring that the dissolved oxygen level did not decrease during deployments. The bottom of the acrylic frame was connected to a drogue. A fine braided line attached the top of the acrylic frame connected first to an intermediate submerged float and then to a surface buoy (Fig. 1b). This reduced tension on the braided line and minimized wind forcing on the chamber. The chamber in which cod larvae swam was 40 cm wide (diameter) and 15 cm deep. The behavior of cod larvae in the chamber was recorded using a GOPRO HERO 7 camera placed at the top of the acrylic frame, looking downwards into the behavioral chamber (Fig. 1b). The device is also outfitted with an AMX-Remora hydrophone, a *HOBO Pendant®* Temperature/Light 64 K Data Logger—UA-002-64, a CTD data logger (DST CTD, *Star-Oddi*, Iceland) and three analog compasses. The float at the top was equipped with a *Locosys* gw-60 GPS, which recorded the location of the drifting chamber every second throughout the experiment.

The chambers were designed to eliminate potential physical damage or stress for fish larvae that could occur because of waves at the surface at the time when larvae were released into the chambers from the boat. The chamber prevents larvae from being affected by surface waves thanks to a foldable and removable rigid chamber wall. While the acrylic frame was held suspended next to the boat with a hydraulic crane, the removable wall was placed around the mesh chamber wall, and the chamber was filled with seawater. Larvae were then released into the chamber, which was then closed with an acrylic lid. The chamber was then lowered into the water until it was fully submerged. Once submerged, the foldable rigid wall was removed from the chamber remotely by using a rope that was connected to it. Once the foldable wall was removed, the drifting chamber was released from the boat and the larvae drifted in the chamber enclosed only by a mesh wall.

For each deployment, two larvae were released into the chamber and their behavior was recorded for 15 min while the chamber was drifting. As two chambers were deployed simultaneously (for most tests), it was possible to record the behavior of up to four larvae simultaneously. After the 15 min deployment ended, the chamber was retrieved, the larvae were replaced with two new larvae, and another test was performed.

Each of the two larvae in the chamber was considered an independent observation, as larvae do not shoal at this stage of development. This protocol was repeated for a total of 90 cod larvae, but one larva escaped during one of the tests. Thus, observations were collected for a total of 89 cod larvae (*N* = 89). Half of the larvae were tested with the sound projector off (Control group; *N* = 44), and half were tested with the sound projector on (Exposed group; *N* = 45). Deployments of chambers with Control and Exposed larvae were alternated throughout the experiment. As described above, the larvae of the Exposed group were exposed to a signal of 100 Hz that was transmitted for the whole 15 min period of each deployment.

### Quantifying orientation and swimming behavior and statistical analysis

We considered the first 5 min of each 15 min deployment as an acclimation period. Thus, larval swimming behavior during the last 10 min was analyzed. All of the videos were processed using the DISCR tracking procedure, utilizing R and a graphical user interface provided by ImageJ software^38,40^. The open-source code utilized (GNU General Public License v3.0) is available at the web page (https://github.com/jiho/discr).

We tracked the position of each fish, every second (600 data points per each larva). See Fig. S1 for a detailed flow chart of all the steps in the analysis. We considered each position of the fish with respect to the center of the arena (in degrees) as a single data point, which we call a bearing. Afterward, we corrected the bearings with respect to the magnetic north using analog compasses. If the frequency distribution of the 600 bearings of one individual larva was significantly different from random (Rayleigh’s *P* < 0.05), we considered it as evidence of orientation, and we used the mean individual bearing as the orientation direction of the larva. The null hypothesis of Rayleigh’s test is the uniformity of the distribution of the bearings. The alternative hypothesis is a unimodal or a Von Mises distribution (which is narrower than the unimodal) with a mean angle, representing the mean direction of the larva. This test is particularly recommended for >30 data points (here there are 600 per fish larva)^44^. The next step of the analysis was to evaluate whether the larvae of each experimental group (Control; Exposed) were swimming toward a common direction (whether they had a common orientation direction; Fig. S1). To explore that, we used Rayleigh’s test of uniformity applied to all of the mean individual bearings of all of the larvae from each of the experimental groups as data points (*N* = 44 larvae tested for the Control group; *N* = 45 larvae for the Exposed group). If Rayleigh’s *P* was <0.05, we interpreted that as evidence that the treatment group of larvae was showing a common orientation, with the mean as the overall common direction of the group. The orientation direction of the Exposed larvae towards the direction of the sound projector was tested by using Rayleigh’s test with the alternative hypothesis of a unimodal distribution with a specified mean direction (in this case, the direction of the sound projector is 0°). Possible effects on orientation accuracy of the variation in delivered sound intensity was tested using the Spearman correlation test.

The median and maximum routine swimming speed of cod larvae between the Control and Exposed group was compared. The routine swimming speed is the spontaneous speed of undisturbed larvae over a period of time that varies depending on the study (usually several minutes)^42,45^. Differences in body length, swimming speed, and proportion of turns greater than 45° between Control and Exposed groups were tested using the nonparametric Wilcoxon test as data were not uniformly distributed. The frequency distribution of the average turning angle was compared between groups using the Kolmogorov-Smirnov test.

### Acoustic processing

Using the AVS GeoSpectrum M20-105, the acoustic directionality (bearing) is determined by applying the active intensity method^46–48^. This method computes the directionality at a sample time and frequency. It is used here to estimate the direction of the source of specific acoustic frequencies. The acoustic bearing (angle of the incoming acoustic wave with the $x$-channel of the AVS pointing to the magnetic north) is used to assess the azimuth direction of the sound between the AVS and a strong acoustic source (the C-BASS). This was performed to verify that the main direction of the 100 Hz acoustic wave in the fjord was towards the C-BASS acoustic projector. Therefore, only the X and Y channels of the AVS were used in the data processing, including the absolute particle velocity of the XY plane. The bearing estimations were validated during a test performed with the AVS laying on the sea floor (at a fixed position relative to the sound source). The direction of the acoustic wave produced by the sound source was validated using the GPS coordinates of the AVS and the acoustic projector. The processing parameters provided in the figure captions were calculated following the guidelines of Robinson, Lepper, and Hazelwood (2014). The bearing and the SPL of 90–110 Hz were calculated at a sampling rate of 1 s.

### Statistics and reproducibility

Differences in SPL and acoustic particle velocity between experimental groups and the drifting chambers were tested with the nonparametric Wilcoxon test. Differences in median and maximum swimming speed between experimental groups were tested with the nonparametric Wilcoxon test. Orientation directionality was tested with Rayleigh’s test for circular data. Orientation towards the predetermined direction of the sound source was tested with Rayleigh’s test for circular data with a specified mean direction. The statistical details on specific methods are described in the results, in the corresponding sections.

### Ethical statement

The Austevoll Research Station has a permit to operate as a Research Animal facility for fish (all developmental stages), under code 93 from the national Institutional Animal Care and Use Committee (IACUC); NARA. We did not require specific approval for these experiments because they are behavioral observations of a non-intrusive potential stimulus.

### Reporting summary

Further information on research design is available in the Nature Portfolio Reporting Summary linked to this article.

## Supplementary information

Supplementary Information

Description of Additional Supplementary Files

Supplementary Data 1

Reporting Summary

## Data Availability

Source data are available in Supplementary Data 1 and Supplementary Information.

## Code availability

The code is available from the corresponding author upon request.
