# Supplementary material for: Atlantic cod (Gadus morhua) larvae are attracted by low-frequency noise simulating that of operating offshore wind farms
Source: Commun Biol. 2023 Apr 12;6:353. doi: 10.1038/s42003-023-04728-y (PMC10097813; doi:10.1038/s42003-023-04728-y)
Supplement: Supplementary file 1 — Supplementary Information [file 42003_2023_4728_MOESM1_ESM.pdf]

Supplementary Information

Atlantic cod (*Gadus morhua*) larvae are attracted by low-frequency noise simulating that of operating offshore wind farms

Alessandro Cresci[1,*,a], Guosong Zhang[1,a], Caroline M.F. Durif[1], Torkel Larsen[1], Steven Shema[1], Anne Berit Skiftesvik[1] & Howard I. Browman[1]

[1]Institute of Marine Research, Austevoll Research Station, Sauganeset 16, N-5392 Storebø, Norway

[a]These authors contributed equally to the work
*Corresponding author: Alessandro Cresci,
Institute of Marine Research, Austevoll Research Station, Sauganeset 16, N-5392 Storebø, Norway
author email: alessandro.cresci@hi.no

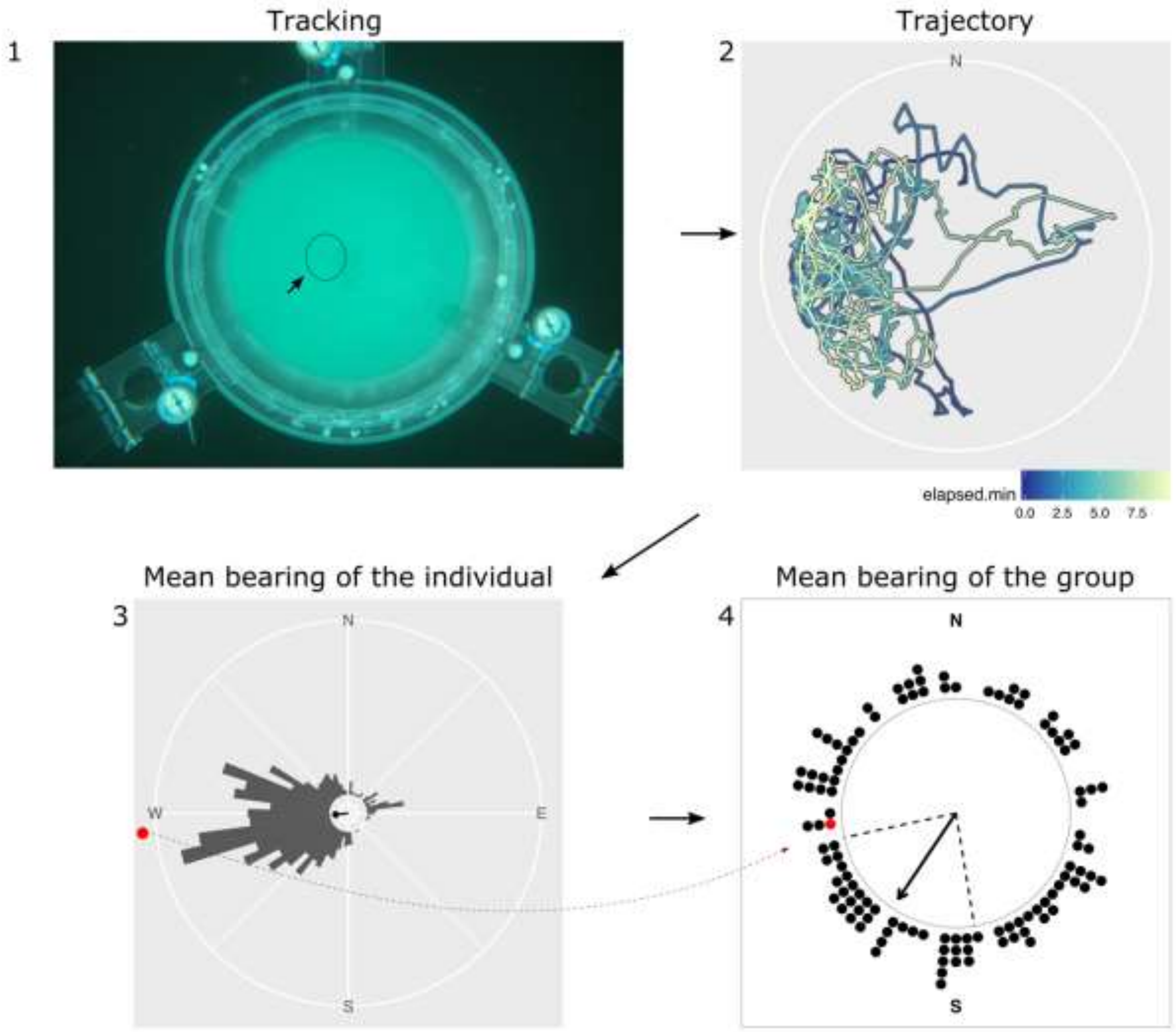

Figure S1 – Description of the analysis of the data collected with the drifting chambers. 1: The first step is to collect data on the position of the larvae in the chamber using a tracking procedure conducted on the videos recorded *in situ* during the deployments. The position of each larva is tracked every second for 10 minutes (i.e. 600 data points are collected per each fish tested in the chamber). The photo shows an example of the view of a cod larva (highlighted by a black arrow) swimming in the behavioral chamber. **2**: The trajectory of the larva is calculated from the datapoints collected through the video tracking. **3**: The angle of each of the 600 data points with respect to the magnetic North and the center of the chamber is considered as a bearing. Because the chamber is allowed to rotate, bearings are corrected using the digital compass that records the difference between the orientation of the camera and the magnetic North. The mean orientation of the larva is assessed by applying Rayleigh's test of uniformity on the 600 bearings. If the outcome of the statistical test is significant ($p < 0.05$), the mean bearing (red circle in this example) is considered as the preferred orientation direction of the larva. **4**: The last step of the analysis is performed on all of the preferred orientation directions of the larvae from the same experimental group (i.e. all the Control or Exposed larvae). The mean orientation directions of the larvae are grouped and the Rayleigh's test is applied. Through this step we assess whether the larvae had the tendency to orient towards a common direction. In this panel of the figure the red circle corresponds to the mean orientation of the larva used as an example in the previous panel # 3. In this hypothetical example, larvae had the tendency to orient SW (direction indicated by the black arrow, with the dashed lines indicating the 95% confidence intervals).

Table S1 – Experiment log and orientation of the cod larvae (*Gadus morhua*) tested relative to the magnetic north. Date = date of the test; **larva_id** = id of each fish larva tested; **chamber** = id of the chamber where each larva was tested; **sound_transmission** = id of each transmission of the 100 Hz signal produced by the C-BASS sound projector; **treatment** = treatment group. Control larvae were not exposed to the 100 Hz sound (C-BASS was switched OFF), exposed larvae were observed when the C-BASS was switched ON; **mean_angle** = mean orientation angle (relative to the magnetic north) of each larva calculated from the video tracks of the larvae. The tracking was performed at a frequency of 1/s. The mean orientation angle is reported for larvae that displayed a preferred orientation direction; **r** = Rayleigh's r value indicating the concentration of the fish larva tracks in a specific section of the chamber (accuracy of the orientation, value ranging 0-1). There was no chamber effect on orientation within the Control group ($F < 0.01$, $p = 0.98$), within the Exposed group ($F = 0.08$ $p = 0.78$), or within the Exposed group relative to the sound source ($F = 0.52$, $p = 0.47$) (Analysis of Variance for Circular Data, High concentration F-Test).

Date	larva_id	chamber	sound_transmission	treatment	mean_angle	r
14-06-2021	1	A	NA	control	314,10	0,26
14-06-2021	2	A	NA	control	275,35	0,21
14-06-2021	3	B	NA	control	49,46	0,41
14-06-2021	4	B	NA	control	4,05	0,45
14-06-2021	5	A	1	exposed	252,79	0,16
14-06-2021	6	A	1	exposed	326,38	0,10
14-06-2021	7	B	1	exposed	310,09	0,08
14-06-2021	8	B	1	exposed	340,18	0,39
14-06-2021	9	A	NA	control	276,64	0,21
14-06-2021	10	A	NA	control	355,74	0,21

14-06-2021	11	B	NA	control	273,50	0,70
14-06-2021	12	B	NA	control	330,68	0,50
14-06-2021	13	A	2	exposed	337,67	0,18
14-06-2021	14	A	2	exposed	158,14	0,14
14-06-2021	15	B	2	exposed	172,94	0,74
14-06-2021	16	B	2	exposed	279,12	0,57
14-06-2021	17	A	NA	control	274,20	0,34
14-06-2021	18	A	NA	control	283,71	0,07
14-06-2021	19	B	NA	control	284,23	0,64
14-06-2021	20	B	NA	control	1,06	0,58
14-06-2021	21	A	3	exposed	230,51	0,42
14-06-2021	22	A	3	exposed	24,51	0,35
14-06-2021	23	B	3	exposed	330,39	0,46
14-06-2021	24	B	NA	control	154,86	0,31
14-06-2021	25	B	NA	control	23,60	0,35
14-06-2021	26	B	4	exposed	299,83	0,52
14-06-2021	27	B	4	exposed	315,54	0,44
14-06-2021	28	B	NA	control	199,50	0,33
14-06-2021	29	B	NA	control	227,61	0,24
15-06-2021	30	A	5	exposed	110,26	0,09
15-06-2021	31	A	5	exposed	298,20	0,67
15-06-2021	32	B	5	exposed	268,80	0,36
15-06-2021	33	B	5	exposed	149,63	0,21
15-06-2021	34	A	NA	control	252,27	0,27
15-06-2021	35	A	NA	control	274,49	0,21
15-06-2021	36	B	NA	control	193,69	0,06
15-06-2021	37	B	NA	control	120,11	0,44
15-06-2021	38	A	6	exposed	232,72	0,09
15-06-2021	39	A	6	exposed	106,38	0,51
15-06-2021	40	B	6	exposed	-	-
15-06-2021	41	B	6	exposed	21,91	0,42
15-06-2021	42	A	NA	control	102,31	0,22
15-06-2021	43	A	NA	control	83,13	0,73
15-06-2021	44	B	NA	control	104,11	0,38
15-06-2021	45	B	NA	control	195,38	0,34
15-06-2021	46	A	7	exposed	202,20	0,31
15-06-2021	47	A	7	exposed	278,57	0,19
15-06-2021	48	B	7	exposed	155,44	0,50
15-06-2021	49	B	7	exposed	172,50	0,67
15-06-2021	50	A	NA	control	321,28	0,50
15-06-2021	51	A	NA	control	240,98	0,60
15-06-2021	52	B	NA	control	3,27	0,19
15-06-2021	53	B	NA	control	285,10	0,65
15-06-2021	54	A	8	exposed	327,17	0,12
15-06-2021	55	A	8	exposed	279,76	0,06
15-06-2021	56	B	8	exposed	341,60	0,54
15-06-2021	57	B	8	exposed	341,42	0,35
15-06-2021	58	A	9	exposed	218,12	0,50
15-06-2021	59	A	9	exposed	50,42	0,47
15-06-2021	60	B	9	exposed	275,01	0,28
15-06-2021	61	B	9	exposed	37,92	0,26
15-06-2021	62	A	NA	control	245,65	0,58
15-06-2021	63	A	NA	control	245,05	0,72
15-06-2021	64	B	NA	control	8,95	0,57
15-06-2021	65	B	NA	control	352,83	0,70
16-06-2021	66	A	10	exposed	99,45	0,24
16-06-2021	67	A	10	exposed	56,81	0,30
16-06-2021	68	B	10	exposed	106,87	0,53

16-06-2021	69	B	10	exposed	55,42	0,13
16-06-2021	70	A	11	exposed	-	-
16-06-2021	71	A	11	exposed	182,47	0,15
16-06-2021	72	B	11	exposed	341,12	0,57
16-06-2021	73	B	11	exposed	338,00	0,56
16-06-2021	74	A	12	exposed	0,24	0,47
16-06-2021	75	A	12	exposed	348,43	0,41
16-06-2021	76	B	12	exposed	18,85	0,53
16-06-2021	77	B	12	exposed	326,60	0,85
16-06-2021	78	A	NA	control	22,33	0,49
16-06-2021	79	A	NA	control	321,13	0,62
16-06-2021	80	B	NA	control	35,04	0,26
16-06-2021	81	B	NA	control	277,07	0,22
16-06-2021	82	A	NA	control	34,39	0,50
16-06-2021	83	A	NA	control	7,25	0,44
16-06-2021	84	B	NA	control	319,28	0,69
16-06-2021	85	B	NA	control	57,58	0,33
16-06-2021	86	A	NA	control	51,09	0,82
16-06-2021	87	A	NA	control	300,50	0,82
16-06-2021	88	B	NA	control	59,84	0,46
16-06-2021	89	B	NA	control	27,64	0,15

Table S2 – Orientation of the exposed larvae with respect to the sound source. mean_angle = mean orientation angle (relative to the sound projector) of each larva calculated from the video tracks of the larvae and the GPS tracks of the drifting chamber and the sound projector. The angle correction relative to the sound source was performed at a frequency of 1/s. **r** = Rayleigh's r value.

Larva_id	mean_angle	r
5	2,27	0,12
6	359,87	0,11
7	91,35	0,38
8	143,61	0,29
13	133,08	0,20
14	317,84	0,12
15	250,73	0,75
16	354,55	0,59
21	21,94	0,67
22	180,97	0,55
23	242,42	0,47
26	3,47	0,52
27	17,58	0,44
30	279,81	0,09
31	96,66	0,65
32	73,35	0,33
33	311,05	0,20
38	251,99	0,25
39	81,46	0,18
41	122,02	0,23
46	352,36	0,29
47	76,61	0,18
48	302,16	0,46
49	320,52	0,66
54	309,88	0,15
55	245,64	0,17
56	319,24	0,47
57	322,03	0,28

58	33,47	0,43
59	237,05	0,38
60	33,74	0,34
61	172,59	0,28
66	197,10	0,25
67	132,10	0,28
68	176,44	0,45
69	116,67	0,13
71	223,49	0,14
72	3,55	0,58
73	1,16	0,54
74	14,52	0,42
75	4,92	0,36
76	24,51	0,50
77	333,43	0,82
