# Supplementary material for: Atlantic cod (Gadus morhua) larvae are attracted by low-frequency noise simulating that of operating offshore wind farms
Source: Commun Biol. 2023 Apr 12;6:353. doi: 10.1038/s42003-023-04728-y (PMC10097813; doi:10.1038/s42003-023-04728-y)
Supplement: Supplementary file 4 — Reporting Summary [file 42003_2023_4728_MOESM4_ESM.pdf]

nature portfolio

Corresponding author(s): Alessandro Cresci

Last updated by author(s): Oct 10, 2022

Reporting Summary

Nature Portfolio wishes to improve the reproducibility of the work that we publish. This form provides structure for consistency and transparency in reporting. For further information on Nature Portfolio policies, see our Editorial Policies and the Editorial Policy Checklist.

Statistics

For all statistical analyses, confirm that the following items are present in the figure legend, table legend, main text, or Methods section.

n/a	Confirmed	
☐	☒	The exact sample size (*n*) for each experimental group/condition, given as a discrete number and unit of measurement
☐	☒	A statement on whether measurements were taken from distinct samples or whether the same sample was measured repeatedly
☐	☒	The statistical test(s) used AND whether they are one- or two-sided<br>*Only common tests should be described solely by name; describe more complex techniques in the Methods section.*
☐	☒	A description of all covariates tested
☒	☐	A description of any assumptions or corrections, such as tests of normality and adjustment for multiple comparisons
☐	☒	A full description of the statistical parameters including central tendency (e.g. means) or other basic estimates (e.g. regression coefficient) AND variation (e.g. standard deviation) or associated estimates of uncertainty (e.g. confidence intervals)
☐	☒	For null hypothesis testing, the test statistic (e.g. *F*, *t*, *r*) with confidence intervals, effect sizes, degrees of freedom and *P* value noted<br>*Give P values as exact values whenever suitable.*
☒	☐	For Bayesian analysis, information on the choice of priors and Markov chain Monte Carlo settings
☒	☐	For hierarchical and complex designs, identification of the appropriate level for tests and full reporting of outcomes
☒	☐	Estimates of effect sizes (e.g. Cohen's *d*, Pearson's *r*), indicating how they were calculated

Our web collection on statistics for biologists contains articles on many of the points above.

Software and code

Policy information about availability of computer code

Data collection	The data on sound and physical variables were collected with in situ instruments. Data on animal behavior were collected with a publicly available tracking procedure deposited on GitHub: https://github.com/jiho/discr
Data analysis	The data analysis was conducted with RStudio Version 1.3.1056 © 2009-2020 RStudio, PBC. The analysis was performed using open source R packages: "circular"; "discr", "ggplot2". All packages are in the CRAN repository: https://cran.r-project.org/web/packages/

For manuscripts utilizing custom algorithms or software that are central to the research but not yet described in published literature, software must be made available to editors and reviewers. We strongly encourage code deposition in a community repository (e.g. GitHub). See the Nature Portfolio guidelines for submitting code & software for further information.

Data

Policy information about availability of data

All manuscripts must include a data availability statement. This statement should provide the following information, where applicable:

- Accession codes, unique identifiers, or web links for publicly available datasets
- A description of any restrictions on data availability
- For clinical datasets or third party data, please ensure that the statement adheres to our policy

All data in included in the supplementary information

Human research participants

Policy information about studies involving human research participants and Sex and Gender in Research.

Reporting on sex and gender	*Use the terms sex (biological attribute) and gender (shaped by social and cultural circumstances) carefully in order to avoid confusing both terms. Indicate if findings apply to only one sex or gender; describe whether sex and gender were considered in study design whether sex and/or gender was determined based on self-reporting or assigned and methods used. Provide in the source data disaggregated sex and gender data where this information has been collected, and consent has been obtained for sharing of individual-level data; provide overall numbers in this Reporting Summary. Please state if this information has not been collected. Report sex- and gender-based analyses where performed, justify reasons for lack of sex- and gender-based analysis.*
Population characteristics	*Describe the covariate-relevant population characteristics of the human research participants (e.g. age, genotypic information, past and current diagnosis and treatment categories). If you filled out the behavioural & social sciences study design questions and have nothing to add here, write "See above."*
Recruitment	*Describe how participants were recruited. Outline any potential self-selection bias or other biases that may be present and how these are likely to impact results.*
Ethics oversight	*Identify the organization(s) that approved the study protocol.*

Note that full information on the approval of the study protocol must also be provided in the manuscript.

Field-specific reporting

Please select the one below that is the best fit for your research. If you are not sure, read the appropriate sections before making your selection.

☐ Life sciences ☐ Behavioural & social sciences ☒ Ecological, evolutionary & environmental sciences

For a reference copy of the document with all sections, see nature.com/documents/nr-reporting-summary-flat.pdf

Ecological, evolutionary & environmental sciences study design

All studies must disclose on these points even when the disclosure is negative.

Study description	The study explored potential effects of exposure to low-frequency continuous sound (in the intensity range of that produced by wind turbines) on swimming and orientation behavior of cod larvae in situ. For this study, cod larvae were video-recorded while drifting in transparent drifting chambers in a fjord while they were exposed to continuous low-frequency sound reproduced by a sound projector.
Research sample	45 cod larvae (Gadus morhua) were exposed to continuous low-frequency sound at 100 Hz frequency. 44 larvae (controls) were observed in the same conditions but in absence of the low-frequency sound.
Sampling strategy	The sample size is sufficient to identify possible differences in swimming performance and orientation behavior.
Data collection	Acoustic data were collected by hydrophones mounted on drifting chambers and by an acoustic vector sensor that was also drifting. Behavioral data were collected through video-tracking procedures applied on videos of cod larvae swimming in drifting chambers.
Timing and spatial scale	The experiments were conducted between 14-16 June 2021. Tests were conducted at a frequency of 1/15 minutes for approximately 3 hours every day.
Data exclusions	*If no data were excluded from the analyses, state so OR if data were excluded, describe the exclusions and the rationale behind them, indicating whether exclusion criteria were pre-established.*
Reproducibility	The experiment is reproducible with the same instrumentation and experimental animals.
Randomization	Tests on Control and Exposed larvae were alternated throughout the experiment.
Blinding	Video tracking was performed without information on the experimental group to which the larvae that were tracked belong.

Did the study involve field work? ☒ Yes ☐ No

Field work, collection and transport

Field conditions	Bjornafjorden, Norway. The ambient temperature in the fjord at 5 m depth was 13.9 (0.13) □C and the salinity 29.8 PSU
Location	Austevoll (60.090 N, 5.290 E), Norway,
Access & import/export	The location was reached with coastal boats belonging to the IMR Austevoll Research Station. Animals were transported responsibly with regular replacement of water to maintain temperature and oxygen. The Austevoll Research Station has a permit to operate as a Research Animal facility for fish (all developmental stages), under code 93 from the national Institutional Animal Care and Use Committee (IACUC); NARA. We did not require specific approval for these experiments because they are behavioral observations of a non-intrusive potential stimulus.
Disturbance	n/a

Reporting for specific materials, systems and methods

We require information from authors about some types of materials, experimental systems and methods used in many studies. Here, indicate whether each material, system or method listed is relevant to your study. If you are not sure if a list item applies to your research, read the appropriate section before selecting a response.

Materials & experimental systems

n/a	Involved in the study	
☒	☐	Antibodies
☒	☐	Eukaryotic cell lines
☒	☐	Palaeontology and archaeology
☐	☒	Animals and other organisms
☒	☐	Clinical data
☒	☐	Dual use research of concern

Methods

n/a	Involved in the study	
☒	☐	ChIP-seq
☒	☐	Flow cytometry
☒	☐	MRI-based neuroimaging

Animals and other research organisms

Policy information about studies involving animals; ARRIVE guidelines recommended for reporting animal research, and Sex and Gender in Research

Laboratory animals	Atlantic cod larvae (Gadus morhua)
Wild animals	The study did not involve wild animals
Reporting on sex	This information could not be collected as the study was performed on fish larval stages, when sex is not identifiable.
Field-collected samples	This study does not use field collected samples
Ethics oversight	We did not require specific approval for these experiments because they are behavioral observations of a non-intrusive potential stimulus.

Note that full information on the approval of the study protocol must also be provided in the manuscript.
